# Hyperbaric Oxygen Therapy for Severe Pediatric Traumatic Brain Injury: A Secondary Psychometric and Nonparametric Analysis of a Retrospective Case Series

**DOI:** 10.7759/cureus.79777

**Published:** 2025-02-27

**Authors:** Tami Peterson, Sheila Burgin, Robert Sherwin, Frederick Strale

**Affiliations:** 1 Hyperbaric Oxygen Therapy, The Oxford Center, Brighton, USA; 2 Hyperbaric Oxygen Therapy, Wayne State University School of Medicine, Detroit, USA; 3 Biostatistics, The Oxford Center, Brighton, USA

**Keywords:** concurrent validity, cronbach’s alpha, glasgow coma scale (gcs), glasgow outcome scale (gos), hyperbaric oxygen therapy (hbot), hypoxic-ischemic encephalopathy (hie), internal consistency reliability, ischemic stroke, pediatric traumatic brain injury, traumatic brain injury (tbi)

## Abstract

This secondary analysis builds upon the results of a previous study conducted at the Centre of Hyperbaric Medicine, Ostrava City Hospital, Czech Republic, from 2019 to 2023. Statistically and clinically significant patient responses to hyperbaric oxygen therapy (HBOT) in children with brain injuries were reported. The current study aims to replicate and extend this original work through a secondary analysis. By applying psychometric (reliability and validity) and non-parametric inferential techniques, this research seeks to elucidate previously unexplored data dimensions.

The secondary analysis results indicated a moderate level of internal consistency as measured by Cronbach's alpha for the Glasgow coma scale (GCS) across multiple time points (r = 0.719). Due to the skewed nature of the data, non-parametric analyses were necessary. The Glasgow outcome scale (GOS) exhibited strong (r = 0.893) internal consistency. Regarding concurrent validity, robust positive Spearman's correlations (rs = 0.831, p < 0.001, 95% CI (0.615, 0.931)), (rs = 0.826, p < 0.001, 95% CI (0.605, 0.929)), (rs = 0.645, p = 0.002, CI (0.283, 0.846)), (rs = 0.598, p = 0.004, 95% CI (0.211, 0.823)) were found between pre-test and post-test HBOT GCS and GOS scores, highlighting their congruence. However, no significant Spearman correlation (rs = -0.205, p = 0.374, 95% CI (-0.594, 0.262)) emerged between the differences in post-treatment GCS and GOS scores.

When analyzing gender differences, no statistically significant differences (Mann-Whitney U = 43.5, Wilcoxen W = 109.5, p = 0.426) (Mann-Whitney U = 45.0, Wilcoxen W = 111.0, p = 0.512) were observed in GCS and GOS gender outcomes. Similarly, etiological comparisons between traumatic brain injury (TBI) and hypoxic-ischemic encephalopathy (HIE) groups (Mann-Whitney U = 46.5, Wilcoxen W = 82.5, p = 0.910), (Mann-Whitney U = 42.0, Wilcoxen W = 120.0, p = 0.678) did not yield significant etiology differences. Finally, the repeated measures analysis showed a substantial and notable improvement (Wilcoxen Signed Rank Test: z = -2.956, p = 0.003, r = -0.645) in GCS scores following HBOT, indicating and confirming the treatment’s large effect size.

Ultimately, the GCS showed moderate internal consistency across different time points, while the GOS had high internal consistency. Positive Spearman's correlations between pre-test and post-test HBOT GCS and GOS scores supported their concurrent validity. However, no significant relationship was found between post-treatment changes in GCS and GOS. There were no significant gender differences in outcomes or significant differences between TBI and HIE groups. Finally, a repeated measures analysis showed a significant and considerable improvement in GCS scores after HBOT, indicating substantial treatment effectiveness.

## Introduction

Hyperbaric oxygen therapy (HBOT) involves the use of pure oxygen at increased pressure, typically 2-3 atmospheres absolute (ATA), resulting in elevated oxygen levels in the blood (hyperoxemia) and tissues (hyperoxia). The increased pressure and oxygen bioavailability might be related to many applications, particularly in hypoxic regions, which also exert antimicrobial, immunomodulatory, and angiogenic properties. Equally, the use of HBOT for ischemic stroke and brain injury is an interesting point of study. Different studies have demonstrated the importance of this procedure as a prophylactic approach for sequestration of inflammation inherent in stroke and traumatic brain injury (TBI), preventing neuronal death [1-3]. Other uses, such as brain preconditioning before stem cell transplantation, have also been explored [1,3]. The importance of the efficacy and safety of HBOT in these conditions remains to be fully elucidated, although some basic and clinical research has shown encouraging results [1,4]. 

HBOT for TBI 

Peterson et al. commented that HBOT is proposed as a potential treatment for TBI. This therapy combines increased atmospheric pressure with high oxygen concentrations, allowing more oxygen to dissolve into the blood. This elevated oxygen level may enhance cognitive function and support the body’s natural healing processes, alleviating symptoms. The dissolved oxygen can penetrate deeper into injured brain tissues, promoting recovery. As a result, cognitive abilities may improve, and symptoms may be reduced [5,6]. 

HBOT has been studied as a treatment for both TBI and post-traumatic stress disorder (PTSD), with administration ranging from three to 71 months post-injury for mild TBI (mTBI) and within 24 hours for moderate to severe cases. Some benefits have been observed in mTBI patients [7]. A systematic review of 42 studies yielded controversial results concerning the efficiency of HBOT in various neurological conditions with cognitive disturbance outcomes [8]. 

A separate analysis examined the effectiveness of HBOT in treating persistent post-concussion syndrome (PPCS) following mTBI. Patients who received 40 HBOT sessions at 1.5 ATA showed significant cognitive and symptomatic improvements [9]. Another randomized controlled trial (RCT) study concluded that early adjuvant HBOT using 1.4 ATM with one session of one hour daily for 10 days among adults sustaining moderate TBI significantly improves GCS at 10 days. Early adjuvant HBOT is also associated with significantly improved Glasgow outcome scale-extended (GOS-E) at three months post-injury compared to standard of care alone [10]. 

In a retrospective study of patients with chronic neurocognitive impairments from TBI, participants underwent 60 HBOT sessions. Pre- and post-treatment cognitive tests and brain perfusion magnetic resonance imaging (MRI) revealed a notable increase in cerebral blood flow and significant cognitive improvements, particularly in information processing, visual-spatial skills, and motor functions. HBOT may stimulate cerebral angiogenesis, enhancing blood flow to damaged brain areas even years after injury [11]. 

A systematic review reported that HBOT is utilized in multiple pathologies but has demonstrated promising results in the preclinical and clinical trial environment. HBOT ameliorates cerebral edema, intracranial pressure elevation, apoptosis, and neuroinflammation while increasing oxygen delivery to the brain and cerebral glucose utilization. Herein is a review of the existing clinical research examining the use of HBOT in attenuating TBI symptoms and physical manifestations [11]. 

A study at the Jupiter Medical Center between 2012 and 2019 (n=18) found that HBOT was effective in improving physical, cognitive, and single-photon emission computed tomography (SPECT) outcomes for the majority of patients who underwent HBOT, with significant improvements in the Trail Making Test and Veterans Affairs Electronic Care Information (VAECI) scores after HBOT. SPECT imaging also demonstrated increased cerebral blood flow and brain metabolism among our patients after HBOT sessions compared to baseline [12]. 

Wang et al. reported that the HBOT group has significantly improved oxidative stress concentrations of glutathione (GSH) and thiobarbituric acid reactive substances (TBARS) after HBOT and persisted for at least eight weeks. However, HBOT did not improve plasma soluble intercellular adhesion molecule-1 (sICAM-1) and soluble vascular adhesion molecule-1 (sVCAM-1) levels [13]. 

HBOT for HIE

Hypoxic-ischemic encephalopathy (HIE) is a brain injury caused by a lack of oxygen (hypoxia) and blood flow (ischemia) to the brain. It is most associated with newborns who experience complications during birth. However, HIE can also occur in older children and adults. This condition can result from severe respiratory distress, such as near-drowning, choking, or cardiac arrest, which leads to a lack of oxygen and blood flow to the brain [14]. 

Sanchez-Rodriguez and Lopez found that when HBOT is administered promptly, it can promote the survival of the penumbra, modulate the cytokine storm, modify inflammatory cascades, restore mitochondrial function, inhibit apoptosis, reinstate cellular communication and cytoskeleton function, reinstall the functioning of the kinase system, reduce cytotoxic and tissue edema, promote microcirculation, and provide an antioxidant effect [15]. All these secondary mechanisms aid in saving, rescuing, and protecting marginal tissue. When used promptly, HBOT is a non-invasive adjunct treatment that can preserve the marginal tissue affected by ischemia and hypoxia, meet the metabolic needs of the penumbra, reduce inflammatory cascades, prevent the extension of the damaged tissue, and modulate ischemia-reperfusion injury [15]. 

Gong et al. commented on the results of their meta-analysis with 46 clinical RCTs, including 4,199 patients with neonatal HIE. The results indicated that HBOT significantly improved the total efficiency (TEF) of treatment for neonatal HIE patients (odds ratio (OR) = 4.61, 95% confidence interval (CI) (3.70, 5.75), p < 0.00001) and reduced the risk of sequelae (OR = 0.23, 95% CI (0.16, 0.33), p < 0.00001) and the neonatal behavioral neurological assessment (NBNA) scores (mean difference (MD) = 4.51, 95%CI (3.83,5.19, p < 0.00001)). Considering the effectiveness of HBOT neonatal HIE, this meta-analysis suggested that HBOT can be a potential therapy for the treatment of neonatal HIE. Due to the heterogeneity of study protocol and the fact that the patient selection is only from China, more research is needed before this therapy can be widely implemented in the clinic [16]. 

Mielecki, Godlewski, and Salinska reviewed 10 clinical studies on antioxidant defense, apoptosis, and stem cell proliferation in rats and human neonates. In the studies in which HBOT was used, clinical improvement in the condition of newborns suffering from HIE was assessed using multiple methods, such as electrocardiogram (ECG) and transcutaneous oxygen monitoring (TCOM) during HBOT sessions, outcome indicators such as toxicity equivalence factor (TEF), i.e., clinical symptoms, signs, and craniocerebral computed tomography, neuronal sequelae, and the NBNA score. The accumulated results of these studies indicate that the benefits of HBOT were obvious [17]. 

Original study overview 

Hajek, Jor, Tlapak, and Chmelar presented a retrospective case series of children with brain injuries treated with HBOT at the Centre of Hyperbaric Medicine in Ostrava, Czech Republic, from 2019 to 2023. Twenty-one children were treated, with a mean age of 6±4.6 years. 57% had TBI, 38% had HIE, and 5% had an ischemic stroke. The average initial Glasgow coma scale (GCS) [18-20] score at admission was 3.3±0.9 [18]. Pre-HBOT, the GCS was 10.7±3.7, improving to 12.3±3.4 (p=0.004) post-HBOT. The Glasgow outcome scale (GOS) [18,21,22] showed improvement from 3.3±0.8 pre-HBOT to 3.9±1.1 (p<0.001) post-HBOT. Regarding HBOT responses, 33% of patients had a large clinically significant reaction (CSR), 39% had a partial response in the form of a minimally important difference (MID), and 28% had no response. Earlier initiation of HBOT (within four weeks of injury) showed better outcomes (p=0.02). The study demonstrated the effectiveness of HBOT in improving clinical outcomes for brain injury patients. Starting HBOT within four weeks post-injury led to better patient responses. HBOT is feasible and effective for pediatric patients in large regional hyperbaric centers [18]. 

Rationale for secondary analysis 

Hajek, Jor, Tlapak, and Chmelar investigated the effectiveness of HBOT in children with traumatic or non-TBIs. They aimed to evaluate whether HBOT could help mitigate disability symptoms and improve clinical outcomes, measured primarily by GCS and GOS [18].

This secondary analysis aims to replicate and extend their original work. This study seeks to elucidate previously unexplored data dimensions by applying psychometric and non-parametric techniques. In doing so, it aims to uncover novel insights, critically extend the robustness of the original findings, and explore additional relationships within the data that were not addressed in the original study. By employing these complementary analytical approaches, this research aims to contribute a more comprehensive understanding of the dataset, enhancing its scientific value and broader applicability within the field. 

Research questions 

The original analyses were conducted with pre-test and post-test HBOT GCS and GOS data, spawning further research questions. For the existing HBOT GCS and GOS data, what is the internal consistency reliability estimate using Cronbach’s alpha? What are the validity estimates for HBOT GCS vs GOS? What are the gender differences between HBOT GCS and GOS? What are the etiology (TBI vs. HIE) differences between HBOT GCS and GOS regarding gender? Are there statistically significant differences between initial GCS, pre-HBOT GCS, and post-HBOT GCS? 

General objectives 

This research will offer new insights into the reliability, validity, and temporal effectiveness of HBOT on severe TBI and HIE children by comparing the GCS and GOS scores at different stages. Understanding these changes will help quantify HBOT's direct impact over time, an area underrepresented in the current body of knowledge. By demonstrating statistically significant improvements, or lack thereof, this study will reinforce or challenge the assumptions about HBOT’s role in neurological recovery, adding clarity to ongoing debates about its efficacy. 

Objective 1: assess the internal consistency reliability of the existing HBOT GCS and GOS data using Cronbach’s alpha 

This study will provide empirical evidence for the internal consistency of HBOT outcomes measured by the GCS and GOS. While both GCS and GOS are widely used, few studies have examined the reliability of these measures, specifically in the context of HBOT data. By calculating Cronbach’s alpha, the study will ensure the consistency and robustness of these scales for evaluating treatment effects, thereby filling a critical methodological gap and enhancing the precision of HBOT efficacy assessments. 

Objective 2: evaluate the concurrent validity of the HBOT GCS in comparison to the GOS 

This research will provide insight into the concurrent validity between GCS and GOS when applied to HBOT patients. Current literature often treats these scales as interchangeable, but their specific utility in measuring outcomes post-HBOT remains underexplored. By directly comparing the two, this study will clarify how these tools correlate and suggest which might be more suitable for specific HBOT applications. This can refine clinical assessments and improve the selection of appropriate outcome measures in HBOT research. 

Objective 3: analyze the gender differences in outcomes between HBOT GCS and GOS 

Gender differences in response to HBOT and brain injury outcomes are sparsely addressed in the literature. This study will identify potential disparities in treatment efficacy between men and women, contributing valuable gender-specific data. Such insights could lead to more personalized and effective treatment protocols, filling a crucial gap in both HBOT research and broader medical practices that often overlook gender as a significant factor in treatment outcomes. 

Objective 4: explore the gender differences in HBOT GCS and GOS based on etiology (TBI vs. HIE)

By examining how HBOT treatments with TBI and HIE affect GCS and GOS scores, this study will understand how different brain injuries respond to HBOT. Furthermore, by analyzing these differences across genders, the study will uncover whether certain etiologies lead to better or worse outcomes for specific genders. This fills a dual gap in the lack of detailed etiology-specific HBOT outcomes and the intersection between gender and etiology, potentially leading to more tailored therapeutic approaches based on injury type and patient gender. 

Objective 5: determine statistically significant differences between (a) pre-HBOT vs. post-HBOT GCS and (b) pre-HBOT vs. post-HBOT GOS

This objective will offer new insights into HBOT's temporal effectiveness by comparing the GCS and GOS scores at different stages: pre-treatment and post-treatment. Understanding these changes will help quantify HBOT's direct impact over time, an area underrepresented in the current body of knowledge. By demonstrating statistically significant improvements, or lack thereof, this study will reinforce or challenge the assumptions about HBOT’s role in neurological recovery, adding clarity to ongoing debates about its efficacy. 

Overall contribution

This secondary analysis addresses several gaps in the literature surrounding the use of HBOT for brain injury recovery, particularly about the measurement tools (GCS and GOS), gender, and etiology. The findings will (1) establish more reliable and valid assessment methods for HBOT outcomes, (2) highlight the importance of gender and etiology in HBOT efficacy, potentially leading to more personalized treatment, and (3) provide a clearer understanding of the temporal effects of HBOT on brain injury recovery, which could inform clinical practices and improve patient outcomes. Bridging these gaps will enhance methodological rigor and contribute to the growing evidence base for HBOT’s use in TBI recovery, paving the way for future research and more nuanced clinical applications. 

## Materials and methods

Original study design

A retrospective case series was conducted at the Centre of Hyperbaric Medicine Ostrava City Hospital, Czech Republic, using data collected over five years (2019-2023). Both TBI and HIE patients after cardiac arrest were included. Once they were medically stable, patients were treated in the subacute to early chronic phase of their injuries (generally two to three weeks post-injury). The patient population included children with severe brain injuries (low initial GCS values) from multiple regions in the Czech Republic [18]. Exclusion criteria included conditions like carbon monoxide poisoning, cancer, severe organ failure, and any condition necessitating mechanical ventilation. Pediatric or neurosurgical specialists made referrals; the hyperbaric center physicians confirmed suitability for HBOT. Each child’s condition was stabilized in standard or intensive care units before transfer to the hyperbaric center [18].

HBOT treatment protocol

A specialized multiplace hyperbaric chamber, pressurized to 2.0 ATA, delivered HBOT sessions. During each session, the patients were instructed to breathe pure oxygen for 75 to 80 minutes, interspersed with two short breaks during which the patients breathed ambient air at chamber pressure. These “air breaks” helped reduce the risk of oxygen toxicity and offered relief from prolonged high-concentration oxygen exposure. An initial 10 to 15 sessions were typically prescribed, although the total number could be modified based on each patient’s clinical response and ability to tolerate the therapy [18].

Before therapy commenced, an ear, nose, and throat (ENT) specialist assessed the patient’s capacity to equalize pressure in their middle ear, often described as “ear clearing” or “ear ventilation.” If this evaluation indicated potential difficulties (e.g., compromised Eustachian tube function), minor interventions such as paracentesis (creating a small opening in the eardrum) could be considered to safeguard against barotrauma and pain. Throughout each HBOT session, a pediatric nurse or physician remained inside the multiplace chamber to closely observe all patients for any signs of discomfort, anxiety, or physiological complications, ensuring that any adverse events were identified and addressed promptly [18].

Monitoring for adverse effects was carried out continuously. These potential side effects could range from mild ear pressure discomfort to more serious problems such as sinus issues, oxygen toxicity, or complications related to existing medical conditions. Should any adverse event arise, whether it required a modification in the treatment protocol, additional medication use, or even a discontinuation of therapy, it was thoroughly documented to track safety and treatment effectiveness. This systematic approach to vigilance and documentation enabled the medical team to adapt HBOT sessions to individual needs and foster a safer treatment environment for all participants [18].

Outcomes

Outcome assessments included the GCS [19,20] and the GOS [21,22] to objectively measure consciousness and functional status. GCS ranges from 3 (deep coma) to 15 (normal consciousness), while GOS ranges from 1 (death) to 5 (mild residual impairment). The initial GCS was measured immediately after injury (hospital admission). Pre- and post-HBOT GCS and GOS were assessed around the start and conclusion of the HBOT course. Improvement was measured by a Large Clinically Significant Response (CSR): ≥2 point gain on GOS, or ≥1 point gain on GOS plus ≥3 point gain on GCS. MID was measured by a ≥1 point gain on GOS and/or ≥1 point gain on GCS. The Overall Response Rate was the combined CSR and MID. No response was defined as no improvements in GCS or GOS.

Data analysis and ethical considerations

Patient demographics, injury severity, time from injury to HBOT, number of sessions, and medication details were extracted from medical charts into Microsoft Excel (Microsoft Corporation, Redmond, Washington, United States). One member of the Czech team gathered the data from the medical charts. Another member was responsible for processing the supplied data and files into tables, performing statistical analysis, and writing the first draft of the manuscript. Statistical tests included the Wilcoxon signed-rank test, which compares pre- and post-HBOT GCS and GOS. Pearson Chi-Square was used to explore relationships between the time to HBOT initiation and response rates (with p < 0.05 considered significant).

Ethical considerations included formal ethical approval, which was waived for this retrospective design under Czech regulations. Parental informed consent for HBOT included permission to analyze data anonymously for research.

Data analysis for secondary analysis

The statistical analyses were conducted using IBM SPSS Statistics version 29.0 IBM Corp., Armonk, US). Cronbach's Alpha coefficients were computed to assess the internal consistency reliability of the variables, namely initial GCS, pre-HBOT GCS, post-HBOT GCS, pre-HBOT GOS, and post-HBOT GOS.

Due to the skewed nature of the data, non-parametric methods were employed to evaluate concurrent validity. Specifically, Spearman’s rank correlation coefficient was used to assess the relationships between the following variable pairs: pre-HBOT GCS and pre-HBOT GOS, post-HBOT GCS and post-HBOT GOS, pre-HBOT GCS and post-HBOT GOS, post-HBOT GCS and the difference in GOS scores (HBOTGOSDIFF), and the difference in GCS scores (HBOTGCSDIFF) and HBOTGOSDIFF. Additionally, gender-based differences in HBOTGCSDIFF and HBOTGOSDIFF were analyzed using the Mann-Whitney U test, as well as etiological differences (TBI vs. HIE) for these same variables. To further evaluate HBOT's pre-test and post-test effects on GCS, a repeated measures analysis was conducted using the Wilcoxon signed-rank test, comparing pre-HBOT GCS and post-HBOT GCS scores. All tests were conducted at an alpha level of 0.05. The null hypothesis was rejected if the p-value was less than 0.05, and statistical significance was inferred.

## Results

Objective 1: Cronbach’s alpha analysis

Table 1 below presents the results of the Cronbach's alpha analysis. Both measures show acceptable to high reliability. The GOS scale (r = 0.893) exhibits stronger consistency than the GCS measures (r = 0.719), meaning GOS scores are more stable and dependable over time. The difference in reliability may suggest that GCS scores fluctuate more across time points, whereas GOS scores remain more stable in assessing overall functional outcomes.

**Table 1:** Cronbach's alpha for initial GCS, pre-HBOT GCS, post-HBOT GCS, pre-HBOT GOS, and post-HBOT GOS GCS, Glasgow coma scale; GOS, Glasgow outcomes scale; HBOT, hyperbaric oxygen therapy

GCS or GOS variable	r
Initial GCS, pre-HBOT GCS, and post-HBOT GCS	0.719
Pre-HBOT GOS and post-HBOT GOS	0.893

Objective 2: concurrent validity - GCS vs. GOS

Table 2 below illustrates the results of the concurrent validity analysis for GCS vs. GOS. Pre- and post-HBOT GCS are strongly correlated with respective GOS scores, confirming the concurrent validity of these measures. Initial GCS moderately predicts post-HBOT GOS, though the relationship is weaker. Changes in GCS scores correlate well with changes in GOS, suggesting that improvements in neurological status (GCS) align with better functional outcomes (GOS). Post-HBOT GCS alone does not strongly predict GOS change, implying that other factors contribute to GOS improvements.

**Table 2:** Concurrent validity - GCS vs. GOS GCS, Glasgow coma scale; GOS, Glasgow outcomes scale; GCSDIFF, Glasgow coma scale pre-post difference; GOSDIFF, Glasgow outcome scale pre-post difference; HBOT, hyperbaric oxygen therapy

Comparison	Spearman's rho (rs)	p-value	95% CI
Pre-HBOTGCS - pre-HBOTGOS	0.831	<0.001	0.615 - 0.931
Post-HBOTGCS - post-HBOTGOS	0.826	<0.001	0.605 - 0.929
Pre-HBOTGCS - post-HBOTGOS	0.598	0.004	0.211 - 0.823
Post-HBOTGCS - HBOTGOSDIFF	-0.205	0.374	-0.594 - 0.262
HBOTGCSDIFF - HBOTGOSDIFF	0.645	0.002	0.283 - 0.846

Objective 3: gender differences - GCS vs. GOS 

Table 3 below presents the results of the gender difference analysis comparing GCS and GOS. There is no evidence that gender influences the extent of improvement in GCS or GOS following HBOT. Both male and female participants showed comparable recovery patterns.

**Table 3:** Gender differences - GCS vs. GOS GCSDIFF, Glasgow coma scale pre-post difference; GOSDIFF, Glasgow outcome scale pre-post difference; HBOT, hyperbaric oxygen therapy

Comparison	Mann-Whitney U	Wilcoxon W	p-value
HBOTGCSDIFF	43.5	109.5	0.426
HBOTGOSDIFF	45.0	111.0	0.512

Objective 4: etiology differences - TBI vs. HIE 

Table 4 below presents etiology differences focusing on TBI vs. HIE. Etiology (TBI vs. HIE) does not significantly impact the improvement in GCS or GOS following HBOT. TBI and HIE patients exhibit similar recovery patterns, indicating that HBOT may be equally effective across these conditions.

**Table 4:** Etiology differences - TBI vs. HIE GCSDIFF, Glasgow coma scale pre-post difference; GOSDIFF, Glasgow outcome scale pre-post difference; HBOT, hyperbaric oxygen therapy

Comparison	Mann-Whitney U	Wilcoxon W	p-value
HBOTGCSDIFF	46.5	82.5	0.910
HBOTGOSDIFF	42.0	120.0	0.678

Objective 5: repeated measures - pre-HBOTGCS vs. post-HBOTGCS and pre-HBOTGOS vs. post-HBOTGOS 

Table 5 below looks at the results of repeated measures analysis focusing on pre-HBOTGCS vs. post-HBOTGCS and pre-HBOTGOS vs. post-HBOTGOS. Wilcoxen signed-rank tests suggest that both GCS and GOS scores significantly improved after HBOT, demonstrating positive effects on neurological and functional recovery. The large effect sizes indicate that these improvements are clinically meaningful, reinforcing the effectiveness of HBOT in enhancing recovery.

**Table 5:** Repeated measures - pre-HBOTGCS/post-HBOTGCS and pre-HBOTGOS/post-HBOTGOS GCS, Glasgow coma scale; GOS, Glasgow outcomes scale; HBOT, hyperbaric oxygen therapy

Comparison	z-value	p-value	Effect size (r)	
Pre-HBOTGCS vs. post-HBOTGCS	-2.956	0.003	-0.645	
Pre-HBOTGOS vs. post-HBOTGOS	-3.357	<0.001	-0.732	

## Discussion

Objective 1: internal consistency reliability with Cronbach’s alpha 

Interpretation of Cronbach’s Alpha for Initial GCS, Pre-HBOT GCS, and Post-HBOT GCS (r = 0.719) 

Cronbach’s alpha is a measure of internal consistency reliability, which evaluates how well a set of variables or items measure a single, unidimensional latent construct [23,24]. A Cronbach's alpha of 0.719 for the variables initial GCS, pre-HBOT GCS, and post-HBOT GCS suggests an acceptable and moderate level of internal consistency. This indicates that the variables (initial, pre-HBOT, and post-HBOT) are reasonably consistent in their ability to reflect the underlying construct of cognitive or functional status over time [23-25]. 

Moderate reliability: The value of r = 0.719 falls within the range generally considered moderate, which spans from r = 0.70 to r = 0.80. This suggests that while there is a meaningful degree of consistency between these variables, some degree of measurement error or variability remains. This indicates that the GCS scores before and after HBOT and the initial GCS are not perfectly aligned when measuring the same latent construct. Gujjar et al. also found that the GCS had good (r = 0.815) internal consistency [26]. These results also align with Mahmoud, Mansour, Roshdy, and Srour, who reported r = 0.720 for their Cronbach's alpha finding [27-31].

Temporal and contextual differences: The moderate alpha could be explained by the temporal and contextual nature of the data. Initial GCS is taken before any treatment, while pre-HBOT GCS and post-HBOT GCS capture scores immediately before and after the intervention. The variability in these measures could reflect actual clinical changes in patients' conditions or the sensitivity of the GCS to detect functional shifts post-treatment [1,2,9,18]. 

Clinical relevance: Given the GCS's nature as a tool that measures levels of consciousness and responsiveness, moderate reliability may be acceptable in clinical practice where patient conditions are expected to fluctuate. The moderate alpha does not negate the utility of these variables in clinical settings. Still, it does suggest that they capture slightly different aspects of the patient's condition at various time points, likely reflecting the dynamic nature of recovery or deterioration in patients undergoing HBOT [26]. 

Construct consideration: It is important to consider whether the moderate alpha indicates that the GCS at different time points reflects not just a single, stable construct (such as an overall neurological function) but also the impact of various other factors (such as therapeutic intervention, time-lapse, and patient-specific responses). The slightly lower reliability may suggest that these variables, while related, are influenced by additional dimensions (e.g., treatment effects) that make the GCS scores less homogenous over time [26]. 

Interpretation of Cronbach’s Alpha for Pre-HBOT GOS and Post-HBOT GOS (r = 0.893) 

In contrast, Cronbach’s alpha of 0.893 for pre-HBOT GOS and post-HBOT GOS indicates a high internal consistency reliability. This suggests that the two GOS scores, which reflect functional outcomes before and after HBOT, are highly consistent in measuring the same underlying construct, likely the patient’s overall functional recovery or neurological status. This compares with Mahmoud, Mansour, Roshdy, and Srour, who reported r = 0.872 for their Cronbach's alpha finding [27,31].

High reliability: A Cronbach’s alpha of 0.893 is typically considered excellent [23,24], suggesting that the pre-HBOT GOS and post-HBOT GOS measures are almost perfectly aligned in capturing the underlying construct of patient outcomes. This high degree of internal consistency implies that these two variables measure very similar dimensions of patient status despite being collected at different time points relative to the HBOT intervention [23,24].

Measurement precision: The high alpha indicates that these GOS scores have low measurement error, meaning that changes between pre-HBOT and post-HBOT GOS are likely due to real differences in patient outcomes rather than variability in the measurement process. This reliability is particularly valuable in clinical research where precise assessments of treatment efficacy (e.g., improvements due to HBOT) are critical [23,24]. 

Validity of outcome measures: The high internal consistency suggests that the GOS is a robust tool for assessing functional outcomes across time. It indicates that the GOS reliably captures the broader construct of patient recovery or deterioration, making it a valid and stable measure for assessing changes pre- and post-HBOT. Such high reliability enhances the credibility of using the GOS in evaluating treatment interventions like HBOT [21-24]. 

Utility in clinical practice: For clinicians and researchers, a high Cronbach’s alpha for the GOS reinforces the utility of this scale as a consistent measure of patient recovery over time [21-24]. It suggests that the GOS can be confidently used to track outcomes before and after HBOT, providing a clear, reliable indication of patient status and treatment efficacy. 

Dimensional homogeneity: The high alpha implies that pre-HBOT and post-HBOT GOS likely measure a unidimensional construct, perhaps "neurological recovery" or "functional independence." This unidimensionality reflects the GOS’s focus on capturing the global outcome of patients in terms of disability or dependence, with minimal influence from external factors or variability [21-24]. 

Comparative Analysis

The difference in Cronbach's alpha between the two sets of variables is notable. The moderate internal consistency of the GCS measures (0.719) compared to the high consistency of the GOS measures (0.893) can be understood as a reflection of the nature of these scales. The GCS is more sensitive to transient, short-term changes in cognitive and motor functions, which may fluctuate depending on treatment, recovery, or deterioration. Therefore, the moderate internal consistency reflects this variability and responsiveness [19-22]. 

In contrast, GOS measures more stable, long-term outcomes (functional independence and disability), which may lead to its higher reliability. Since functional outcomes tend to change more gradually and consistently, the GOS shows greater homogeneity across time points, hence the higher Cronbach's alpha. This comparison illustrates the trade-offs between scales that measure dynamic, short-term changes (GCS) and long-term, stable outcomes (GOS) [19-22]. 

Cronbach's alpha values of 0.719 and 0.893 provide essential insights into the internal consistency and reliability of the GCS and GOS measures. The moderate alpha for the GCS reflects the dynamic nature of neurological assessment at different time points. In contrast, the high alpha for the GOS underscores its reliability in capturing long-term functional outcomes. Both sets of reliability coefficients offer valuable evidence supporting the use of these scales in their respective clinical and research contexts [27]. 

Objective 2: concurrent validity - GCS vs. GOS 

Pre-HBOTGCS - Pre-HBOTGOS (rs = 0.831, p < 0.001, CI (0.615, 0.931)) 

The Spearman's correlation (rs = 0.831) between pre-HBOT GCS and pre-HBOT GOS is strong and statistically significant, with a p < 0.001, indicating that the likelihood of this correlation occurring by chance is extremely low. The 95% CI of 0.615, 0.931 further supports the robustness of this correlation, with the lower bound still indicating a moderate-strong relationship [28-30]. 

The strength of this correlation signifies a high degree of concurrent validity between the GCS and GOS before HBOT. Given that both scales assess related constructs, neurological functioning, and broader functional outcomes, a strong correlation suggests that patients who score higher on the GCS (indicating better consciousness and motor response) also tend to have better functional outcomes on the GOS [28-30].

The narrow CI (0.615 to 0.931) further underscores that the relationship is statistically stable and clinically meaningful. This association reflects that both tools provide a converging indication of the patient’s initial state before HBOT intervention, reinforcing the GCS and GOS as reliable pre-treatment assessments [28-30]. 

*Post-HBOTGCS - Post-HBOTGOS (rs = 0.826, p < 0.001, CI (0.605, 0.929))* 

The correlation (rs = 0.826) between post-HBOT GCS and post-HBOT GOS is similarly strong and statistically significant with p < 0.001. The CI of 0.605, 0.929 mirrors the stability of this association [28-30]. 

The high correlation between post-HBOT GCS and post-HBOT GOS demonstrates that the changes reflected by these scales after HBOT are consistent and capture improvements in related patient outcomes. This strong correlation post-treatment suggests that improvements in neurological function (as measured by GCS) are reliably paralleled by improvements in broader functional outcomes (as measured by GOS), thereby solidifying the concurrent validity of these measures in capturing treatment effects [1,2,9,18,30]. 

The CI is slightly broader than the pre-treatment correlation, which could reflect greater variability in how patients respond to treatment. However, the strength of the correlation remains, indicating that both measures remain highly consistent even after an intervention like HBOT [28-30]. 

Pre-HBOTGCS - Post-HBOTGOS (rs = 0.598, p = 0.004, CI (0.211, 0.823)) 

This correlation (rs = 0.598) between pre-HBOT GCS and post-HBOT GOS is moderate and statistically significant, with p = 0.004. The CI of 0.211, 0.823 is broader than the previous correlations, suggesting more variability in this relationship [30]. 

rs = 0.598 still supports concurrent validity but suggests that the relationship between initial GCS scores before HBOT and functional outcomes measured by the GOS after HBOT is less direct than correlations within the same time frame (pre-pre or post-post). This is expected because pre-HBOT GCS captures initial cognitive/motor function, while post-HBOT GOS measures broader outcomes post-intervention. The variability in patient recovery post-HBOT could explain why the relationship is not as strong. 

Despite the moderate correlation, the p-value (p = 0.004) and CI suggest a meaningful relationship between pre-treatment neurological function and post-treatment outcomes, indicating that pre-HBOT GCS is still somewhat predictive of long-term recovery. However, other factors may influence the outcome. 

*Post-HBOTGCS - HBOTGOSDIFF (rs = -0.205, p = 0.374, CI (-0.594, 0.262))* 

The correlation (rs = -0.205) between post-HBOT GCS and HBOTGOSDIFF (the difference in GOS scores pre- and post-HBOT) is weak and not statistically significant (p = 0.374). The CI (-0.594, 0.262) crosses zero, suggesting this correlation is not meaningful [30]. 

The weak and non-significant correlation indicates that post-HBOT GCS does not meaningfully correlate with the difference in GOS scores due to HBOT. In other words, the neurological improvement post-treatment, as measured by the GCS, does not strongly predict the amount of change in the GOS score. This lack of a strong relationship may suggest that the constructs of immediate post-treatment neurological function (GCS) and overall functional change (GOS) are not tightly aligned in this context. 

The wide CI (-0.594 to 0.262) includes positive and negative values, highlighting this correlation's uncertainty and potential unreliability. The overlap with zero reinforces the lack of a consistent or reliable association between these variables [30]. 

*HBOTGCSDIFF - HBOTGOSDIFF (rs = 0.645, p = 0.002, CI (0.283, 0.846))* 

This correlation (rs = 0.645) between HBOTGCSDIFF (the difference in GCS scores pre- and post-HBOT) and HBOTGOSDIFF is moderately strong and statistically significant (p = 0.002), with a CI of 0.283, 0.846 [30]. 

The correlation indicates that improvements in GCS due to HBOT are moderately associated with improvements in GOS. This suggests that changes in neurological function (GCS) are reflected in broader functional outcomes (GOS), providing concurrent validity between the two scales when measuring improvements due to the intervention [30]. 

The relationship between the differences in GCS and GOS (HBOTGCSDIFF and HBOTGOSDIFF) suggests that patients who experience greater neurological recovery due to HBOT also tend to show greater improvements in their overall functional status. This validates both scales' utility in tracking the intervention's effects [30]. 

The CI (0.283 to 0.846) suggests a stable and reliable relationship, supporting the conclusion that changes in functional outcomes mirror HBOT-driven changes in neurological status. This reinforces the concurrent validity of these scales in assessing recovery [30]. 

From a concurrent validity perspective, the strong and statistically significant correlations between pre-and post-HBOT GCS and GOS, as well as the changes reflected by these scales, support the idea that these measures are closely related and capture similar aspects of neurological function and recovery both before and after HBOT. The weaker or non-significant correlations (such as post-HBOT GCS and HBOTGOSDIFF) suggest that not all aspects of recovery are tightly coupled, which could reflect the multifaceted nature of recovery following therapeutic interventions like HBOT. Overall, the significant correlations underscore the reliability of GCS and GOS as concurrent measures of patient outcomes in both clinical and research contexts [30].

Objective 3: gender differences - GCS vs. GOS 

*HBOTGCSDIFF: Mann-Whitney U = 43.5, Wilcoxon W = 109.5, p = 0.426* 

The Mann-Whitney U value of 43.5 and Wilcoxon W of 109.5 indicate the rank-sum of the data. However, the p-value of 0.426 suggests no statistically significant difference between the two gender groups regarding their change in GCS scores (HBOTGCSDIFF) following HBOT [32,33]. 

The p-value (p = 0.426) is much higher than the conventional threshold of 0.05, indicating that any observed differences in the HBOTGCSDIFF scores between the two gender groups are likely due to chance. This lack of significance implies that the difference in neurological improvement, as captured by the change in GCS scores before and after HBOT, is not substantial between the two gender groups under comparison [32,33]. 

Wilcoxon W Value

The Wilcoxon W value represents the sum of ranks in one group. Since there is no significant difference, this value primarily supports the non-significant Mann-Whitney U result. Although no CIs are provided directly in the output, the non-significant p-value suggests that the CI for the difference in medians would likely include zero, reinforcing the lack of a meaningful difference between groups [32,33]. 

*HBOTGOSDIFF: Mann-Whitney U = 45.0, Wilcoxon W = 111.0, p = 0.512* 

Similarly, the Mann-Whitney U value of 45.0 and Wilcoxon W value of 111.0 reflect the rank-sum results for the change in GOS scores (HBOTGOSDIFF), with a p-value of 0.512. This also indicates no significant difference between the gender groups regarding changes in GOS scores after HBOT [32,33]. 

A p-value of 0.512 suggests no statistically significant gender difference in functional outcome changes (as measured by the GOS) between the two gender groups following HBOT. This implies that any observed differences in the changes in GOS are not large enough to conclude that the groups differ in how they respond to the treatment [32,33]. 

This finding may suggest that other factors, such as comorbidities or initial severity of the condition, do not substantially influence functional outcome improvement as measured by the GOS or that the sample size lacks the power to detect these differences. It could also imply that more nuanced or condition-specific scales may be necessary to detect subtle differences between males and females [32,33]. 

Although specific CIs are not provided, the non-significant p-value indicates that the CI for the difference in GOS change likely includes zero, reaffirming that there is no meaningful gender difference in median rank changes between the groups [32,33]. 

Objective 4: etiology differences - TBI vs. HIE 

*Mann-Whitney U Test Interpretation From an Etiological Perspective (TBI vs. HIE)* 

The interpretation of the Mann-Whitney U test in the context of etiology, specifically comparing patients with TBI and those with HIE, involves examining the differences in outcomes changes in GCS and GOS scores between these two groups following HBOT. This non-parametric statistical test does not assume a normal distribution and assesses differences in median ranks between the two etiological groups [32,33]. 

*HBOTGCSDIFF: Mann-Whitney U = 46.5, Wilcoxon W = 82.5, p = 0.910* 

The Mann-Whitney U value of 46.5 and the p-value of 0.910 indicate no statistically significant difference between the TBI and HIE groups regarding their change in GCS scores (HBOTGCSDIFF) following HBOT. A p-value of 0.910 suggests that the distribution of GCS changes is nearly identical between the two groups, meaning that both TBI and HIE patients experienced similar levels of neurological improvement as measured by changes in GCS after HBOT [32,33]. 

From an etiological perspective, the absence of a significant difference suggests that the mechanism of brain injury, whether due to TBI or HIE, does not lead to differential outcomes regarding GCS improvement post-HBOT. This could imply that HBOT’s effect on neurological recovery may be independent of the initial cause of brain damage. 

The Wilcoxon W value of 82.5 reflects the sum of ranks for one of the groups and aligns with the lack of statistical significance. Although CIs are not provided, the high p-value strongly suggests that the CI for the difference in median rank changes would likely include zero, reinforcing the absence of a meaningful difference between the TBI and HIE groups [32,33]. 

Clinically, this finding indicates that HBOT may be equally effective for improving neurological status (as measured by GCS) in patients with either TBI or HIE. The result supports HBOT's broad applicability across different etiologies of brain injury, suggesting that the therapy could be used in various clinical contexts without expecting differing outcomes based on etiology alone. 

*HBOTGOSDIFF: Mann-Whitney U = 42.0, Wilcoxon W = 120.0, p = 0.678* 

The Mann-Whitney U value of 42.0 and p-value of 0.678 indicate no significant etiological difference between the TBI and HIE groups regarding their change in GOS scores (HBOTGOSDIFF) following HBOT. The p-value of 0.678 is well above the conventional significance threshold (p = 0.05), suggesting that both groups exhibit similar functional outcome changes as measured by the GOS, with no evidence to support that either TBI or HIE patients respond more favorably to HBOT in terms of functional status improvement [32,33]. 

From the viewpoint of etiology, the non-significant result implies that functional recovery, as measured by the GOS, does not differ significantly between patients with brain injury resulting from TBI versus HIE after HBOT. This highlights that the therapy's effects on overall functional status appear comparable regardless of whether the brain injury was caused by trauma or hypoxia-ischemia. 

The lack of statistical difference suggests that the underlying cause of injury may not influence the degree of functional recovery following HBOT, further supporting the concurrent validity of using GOS as a generalizable measure across different types of brain injury. 

The Wilcoxon W value of 120.0 again reflects the sum of ranks for one of the groups and supports the Mann-Whitney U result. The absence of statistical significance suggests that the CI for the difference between the TBI and HIE groups would likely include zero, reinforcing the conclusion that there is no meaningful difference between groups regarding functional outcome changes [32,33]. 

Clinically, this finding suggests that HBOT does not appear to preferentially benefit one etiological group over the other in terms of functional outcome. This may encourage the application of HBOT as a general therapeutic intervention for brain injuries of different origins without needing to adjust expectations based on etiology. 

From a research perspective, this result may indicate that further investigation is needed into other potential moderating variables (e.g., injury severity and time since injury) that could differentiate the response to HBOT, as etiology alone (TBI vs. HIE) does not seem to account for differences in functional improvement. 

From an etiological perspective, the Mann-Whitney U test results for both HBOTGCSDIFF and HBOTGOSDIFF indicate that there is no statistically significant difference between the TBI and HIE groups in terms of either neurological recovery (GCS) or functional outcome (GOS) improvement following HBOT. The high p-values for both comparisons suggest that the changes in GCS and GOS scores are consistent across etiologies, meaning that HBOT is equally effective for patients regardless of whether their brain injury stems from traumatic or hypoxic-ischemic causes. 

These findings imply that HBOT could be considered a broadly applicable treatment for brain injuries, with concurrent validity supported by the GCS and GOS measures across different etiological groups. Moreover, the lack of significant differences highlights the potential for further research into other factors (beyond etiology) that may influence the effectiveness of HBOT in different patient populations. 

From a concurrent validity perspective, the results of the Mann-Whitney U test for both HBOTGCSDIFF and HBOTGOSDIFF suggest that the groups under study do not exhibit significant differences in either neurological recovery or functional outcome improvement following HBOT. The non-significant p-values for both tests indicate that the observed changes in GCS and GOS scores are consistent across groups, reinforcing the potential utility of these measures in broadly capturing patient outcomes. However, the lack of significant between-group differences could also suggest that more refined instruments or a larger sample size might be needed to detect more subtle effects of HBOT on specific subpopulations. These results support the concurrent validity of the GCS and GOS as measures of general recovery but highlight potential limitations in their sensitivity to between-group differences within the context of HBOT-related changes [32,33]. 

Objective 5(a): repeated measures - pre-HBOTGCS vs. post-HBOTGCS 

*Wilcoxon Signed-Rank Test Interpretation: Pre-HBOTGCS vs. Post-HBOTGCS* 

The Wilcoxon signed-rank test is a non-parametric statistical test used to evaluate whether there is a significant difference between two related samples, in this case, the pre-HBOTGCS and post-HBOTGCS scores. The test compares the medians of the pre-test and post-test GCS scores to determine if there is a significant shift in neurological status following the application of HBOT. 

Wilcoxon Signed-Rank Test Results: z = -2.956, p = 0.003, r = -0.645 (Large Effect). 

The z-score of -2.956 and the associated p-value of 0.003 indicate a significant difference between the pre-HBOTGCS and post-HBOTGCS scores. Since the p-value is below the conventional significance threshold of 0.05, we can reject the null hypothesis and conclude that there is a statistically significant improvement in GCS scores following HBOT. The r = -0.645 indicates a large effect [34,35]. 

This finding, similar to Hajek, Jor, Tlapak, and Chmelar, provides robust evidence that HBOT was effective in improving the neurological status of patients, as indicated by the higher GCS scores post-treatment. The negative z-value further suggests that the rank sum of the negative differences (post-HBOTGCS scores being greater than pre-HBOTGCS scores) was larger, indicating an overall improvement in GCS following HBOT [18]. 

Interpretation From an HBOT Effectiveness Perspective

The significant z-value implies evidence of neurological recovery and a consistent improvement in GCS scores for most patients post-HBOT, which directly reflects the effectiveness of HBOT in promoting neurological recovery. The GCS, which measures levels of consciousness and cognitive function, is a key indicator of neurological health, and its improvement post-HBOT highlights the therapeutic benefits of intervention.

*Effect Size (r) Interpretation for Wilcoxon Signed-Rank Test: Pre-HBOTGCS vs. Post-HBOTGCS* 

The effect size (r = -0.645) represents the magnitude of the difference between the pre-test (pre-HBOTGCS) and post-test (post-HBOTGCS) scores in the Wilcoxon signed-rank test. In this context, effect size measures the practical significance of improving neurological status, as indicated by the change in GCS scores after HBOT. 

The effect size (r = -0.645) is considered large by conventional standards. In effect size interpretation, the following thresholds are often used: small effect: r ≈ 0.10, medium effect: r ≈ 0.30, large effect: r ≈ 0.50 or above. 

Thus, with an effect size of r = -0.645, the difference between the pre-test and post-test GCS scores is substantial, indicating that HBOT had a large and meaningful impact on patients' neurological recovery, as measured by the GCS. The negative sign simply reflects the direction of the test (i.e., post-test scores were generally higher than pretest scores). Still, the magnitude of the effect is crucial for interpretation. 

The large effect size indicates practical significance, namely that the improvement in GCS scores post-HBOT was not only statistically significant (as demonstrated by the p-value) but also clinically meaningful. The observed change in GCS represents a substantial improvement in the patient's neurological condition, suggesting that HBOT facilitated considerable recovery in terms of cognitive and consciousness levels. 

In practical terms, this large effect suggests that the treatment profoundly impacted the patient's neurological outcomes. For clinicians, this implies that HBOT is highly likely to produce significant improvements in patients with brain injuries, as measured by standard neurological scales like the GCS. 

Given that an effect size of 0.645 is large, this suggests that HBOT may provide better-than-average improvements than other standard therapies or interventions for brain injury. When considering the magnitude of change, this effect size implies that HBOT has the potential to outperform many traditional neurological rehabilitation techniques, making it a valuable addition to the therapeutic repertoire for improving outcomes in patients with compromised neurological function. 

*Broader Implications for HBOT Efficacy* 

The large effect size supports the view that HBOT is an efficacious treatment for improving neurological function in patients with brain injuries, whether of traumatic or hypoxic-ischemic origin. Given the strength of this effect, it suggests that HBOT could be a key intervention in the management and rehabilitation of patients with significant brain damage, leading to notable improvements in their recovery trajectory. 

The substantial magnitude of the effect also encourages further research into HBOT and its mechanisms. The high effect size provides a foundation for exploring how and why HBOT is so effective in facilitating neurological healing, potentially spurring additional studies into optimal treatment protocols, dosage, and timing. 

The effect size of r = -0.645 reveals a large and practically significant improvement in neurological function (as measured by the GCS) following HBOT [34,35]. This indicates that the therapy had a substantial impact on patients' recovery, reinforcing the idea that HBOT is a highly effective intervention for enhancing consciousness and cognitive function in individuals with brain injuries. The size of this effect suggests that clinicians can expect considerable gains in patient outcomes when using HBOT, further supporting its widespread adoption in neurological rehabilitation. 

*Broader Implications for HBOT in Clinical Practice* 

The improvement in GCS scores reinforces HBOT's utility as an adjunctive treatment for brain injury, whether traumatic or hypoxic-ischemic. This improvement could be attributed to HBOT’s physiological mechanisms, such as enhancing oxygen delivery to hypoxic brain tissues, promoting cellular repair, reducing cerebral edema, and stimulating neuroplasticity. 

Contextualization Within the Literature

The observed significant improvement in GCS scores aligns with existing research on the neurological benefits of HBOT in patients with brain injuries [1,2,9,18]. Previous studies have documented improvements in cognitive function, consciousness levels, and motor skills post-HBOT, particularly in populations with TBI and stroke. The Wilcoxon signed-rank test results further support the hypothesis that HBOT facilitates neurological recovery, potentially via enhanced oxygen diffusion, reduced ischemic brain damage, and improved metabolic function in compromised neural tissues [1,2,9,18]. 

Summation

The results of the Wilcoxon signed-rank test (z = -2.956, p = 0.003, r = -0.645) strongly suggest that HBOT leads to significant neurological improvement, as evidenced by higher post-HBOTGCS scores compared to pre-HBOTGCS scores. From an HBOT effectiveness perspective, these findings underscore the therapy’s role in enhancing cognitive and neurological recovery in patients with brain injuries. The significant change in GCS scores provides compelling evidence that HBOT may be a valuable intervention for improving consciousness and cognitive function in patients with compromised neurological states, warranting its broader consideration in clinical practice for brain injury rehabilitation. 

Objective 5(b): repeated measures - pre-HBOTGOS vs. post-HBOTGOS 

Wilcoxon Signed-Rank Test Interpretation: Pre-HBOTGOS vs. Post-HBOTGOS

The Wilcoxon Signed-Rank Test is a non-parametric statistical method used to evaluate whether there is a significant difference between two related samples, in this context, the pre-HBOTGOS and post-HBOTGOS scores. By comparing the medians of these two sets of scores, the test determines if there is a meaningful shift in patient outcomes (as measured by the GOS) following the administration of HBOT.

Wilcoxon Signed-Rank Test Results: z = -3.357, p < 0.001, r = -0.732 (Large Effect)

A z-score of -3.357 with a p < 0.001 indicates a statistically significant difference between pre-HBOTGOS and post-HBOTGOS. Since the p-value is well below the conventional significance threshold of 0.05, we can reject the null hypothesis. This finding strongly suggests that HBOT is associated with improved patient outcomes on the GOS. Moreover, the r = -0.732 signifies a large effect size, highlighting the practical importance of these improvements.

The negative z-value indicates that the rank sum of the negative differences (where post-HBOTGOS is higher than pre-HBOTGOS) is greater overall, suggesting that most patients experienced improved GOS scores after undergoing HBOT.

Interpretation From an HBOT Effectiveness Perspective

The significant z-value and large effect size together provide strong evidence that HBOT can promote meaningful improvements in patient outcomes, as reflected by the GOS. An increase in GOS score generally denotes better global recovery and functional status, reinforcing the view that HBOT may enhance neurological and functional recovery processes in affected individuals.

Broader Implications for HBOT Efficacy

The large effect size (r = -0.732) supports the contention that HBOT is an effective therapeutic modality for improving overall outcomes in patients with neurological and functional deficits. This level of effectiveness is consistent with the broader body of research suggesting that HBOT can promote tissue repair, reduce edema, and enhance the body’s capacity for recovery following injury or hypoxic events.

In a practical sense, these findings point to the potential for HBOT to outperform many conventional rehabilitation strategies when it comes to elevating outcome scores for patients with brain injuries or other conditions affecting global function. Consequently, clinicians may consider incorporating HBOT into comprehensive treatment plans to optimize recovery trajectories.

Contextualization Within the Literature

These results align with existing evidence indicating that HBOT can lead to meaningful improvements in global functioning and long-term outcomes. Various studies have reported enhanced cognitive abilities, motor function, and daily living skills in patients undergoing HBOT protocols. The significant jump in GOS underscores the notion that higher oxygen availability in the injured or ischemic tissues can accelerate the healing process and possibly mitigate secondary damage [1,2,9,18].

Summation

The Wilcoxon signed-rank test outcome (z = -3.357, p < 0.001, r = -0.732) demonstrates a significant and large effect of HBOT on patient recovery, as measured by the GOS. Clinically, these results affirm that HBOT may substantially boost functional status and overall outcomes for patients with brain injuries or other conditions affecting neurological function. Given the powerful effect size, clinicians can be encouraged that HBOT could represent a highly beneficial addition to the arsenal of therapeutic interventions aimed at improving patient trajectories in neurological rehabilitation settings.

Limitations

Despite the noteworthy findings regarding internal consistency reliability and the effectiveness of HBOT, several limitations should be acknowledged to contextualize the results and guide future research.

The moderate internal consistency of the GCS measures, with a Cronbach’s alpha of 0.719 for initial GCS, pre-HBOT GCS, and post-HBOT GCS, falls within the moderate reliability range. While this indicates a reasonable level of consistency, it also suggests that measurement variability exists. This could stem from several factors.

GCS scores were assessed at different time points (initial, pre-HBOT, and post-HBOT), potentially capturing fluctuating neurological states rather than a stable construct. Unlike long-term outcome measures, GCS is highly sensitive to acute physiological and neurological changes, which may introduce variability in scores. Also, differences in clinician assessment techniques may have contributed to measurement inconsistencies, affecting reliability.

There are potential ceiling and floor effects in GOS measurements. The Cronbach’s alpha for pre-HBOT GOS and post-HBOT GOS was 0.893, indicating excellent reliability. However, this strong consistency could also suggest that GOS measures may have limited sensitivity to subtle clinical improvements or deteriorations due to restricted response range as GOS is a categorical scale with a limited number of outcome categories, which may not capture the full spectrum of recovery.

There may also be reduced sensitivity to incremental change as patients with minor improvements may not show a change in GOS scores, even if their neurological status has improved meaningfully.

Also, there may be a lack of control for confounding variables. Although improvements in GCS and GOS were observed, the study did not fully account for potential confounders that could influence neurological recovery and functional outcomes, including the baseline severity of injury. Differences in initial injury severity could affect how patients respond to HBOT.

In addition, patients may have received additional therapies (e.g., physical rehabilitation and medication adjustments) that could contribute to observed improvements. Variability in the time elapsed between injury, and HBOT initiation could impact the effectiveness of treatment.

The limited sample size for subgroup analyses relative to the gender and etiology (TBI vs. HIE) comparisons did not reveal significant differences in HBOT outcomes. However, the statistical power of these analyses may have been limited due to the small sample sizes in each subgroup. A larger sample might reveal subtle but clinically meaningful differences in treatment response across gender and etiology. Future studies should consider stratifying data by factors such as age, comorbidities, and injury mechanism to better understand subgroup differences.

Given the reliance on clinical rating scales (GCS and GOS), inherent biases may have influenced the findings. There may have been subjectivity in scoring. Despite standardized guidelines, clinicians may interpret patient responses differently when assigning scores.

Observer expectancy effects, stemming from knowledge of the patient's treatment status, may unconsciously influence scoring, leading to potential overestimation or underestimation of effects. Additionally, the data were collected retrospectively, meaning information may have been inaccurately recorded due to human error, misinterpretation, or transcription mistakes. The study may only include patients with available records, potentially excluding relevant cases. Furthermore, variability in how data were recorded across different providers, institutions, or time periods could affect consistency. As a result, the findings may not be applicable to broader populations if the dataset is derived from a single institution or patient group.

While significant improvements were observed in GCS and GOS post-HBOT, this study does not address whether these gains are sustained over time. Future research should incorporate follow-up assessments in the form of longitudinal studies tracking patients for months or years post-HBOT, which could determine if improvements persist or diminish over time. In addition, quality of life and functional independence measures beyond the GCS and GOS, which incorporate patient-reported outcomes and functional independence assessments, would provide a more comprehensive picture of long-term benefits.

In terms of the generalizability of findings, the applicability of these results to broader populations is uncertain due to the fact that this is a single-center study design. If the study was conducted at a single institution, findings may not be generalizable to different clinical settings with varying patient demographics and treatment protocols. The lack of diverse population representation in terms of certain demographic groups (e.g., older adults, pediatric patients), who were underrepresented, means that the findings may not extend to these populations.

While this study provides compelling evidence of HBOT’s efficacy in improving neurological and functional outcomes, these limitations highlight areas for future research and methodological refinement. Addressing these concerns through larger, multicenter, and longitudinal studies with robust control measures will enhance the reliability and applicability of findings, ultimately informing clinical decision-making and optimizing HBOT utilization.

## Conclusions

This secondary analysis revealed moderate internal consistency for the GCS across different time points, while the GOS demonstrated high internal consistency. Regarding concurrent validity, strong positive correlations were observed between pre- and post-HBOT GCS and GOS scores, supporting their alignment. However, no significant relationships were found between post-treatment GCS and GOS differences. In examining gender differences, no statistically significant variation was detected between GCS and GOS outcomes. Similarly, differences between TBI and HIE groups were not significant. Finally, the repeated measures analysis indicated a significant improvement in GCS and GOS scores after HBOT, reflecting large effect sizes and demonstrating the effectiveness of the treatment. 
