# Correction: Hyperbaric Oxygen Therapy for Severe Pediatric Traumatic Brain Injury: A Secondary Psychometric and Nonparametric Analysis of a Retrospective Case Series

**DOI:** 10.7759/cureus.c242

**Published:** 2025-08-13

**Authors:** Tami Peterson, Sheila Burgin, Robert Sherwin, Frederick Strale

**Affiliations:** 1 Hyperbaric Oxygen Therapy, The Oxford Center, Brighton, USA; 2 Hyperbaric Oxygen Therapy, Wayne State University School of Medicine, Detroit, USA; 3 Biostatistics, The Oxford Center, Brighton, USA

The Conflicts of Interest statement has been updated to declare the following:

- Payment/services info: The Oxford Center provided assistance with content input, data gathering and management, and interpretation.
- Financial relationships: Tami Peterson, Sheila Burgin, Robert Sherwin and Frederick Strale Jr. declare(s) employment from The Oxford Center. 

